# SCLC Treatment in the Immuno-Oncology Era: Current Evidence and Unmet Needs

**DOI:** 10.3389/fonc.2022.840783

**Published:** 2022-04-14

**Authors:** Lorenzo Belluomini, Lorenzo Calvetti, Alessandro Inno, Giulia Pasello, Elisa Roca, Emanuela Vattemi, Antonello Veccia, Jessica Menis, Sara Pilotto

**Affiliations:** ^1^ Medical Oncology, Department of Medicine, University of Verona, Verona, Italy; ^2^ Medical Oncology, San Bortolo Hospital, Vicenza, Italy; ^3^ Medical Oncology, IRCCS Sacro Cuore Don Calabria Hospital, Verona, Italy; ^4^ Department of Surgery, Oncology and Gastroenterology, University of Padua, Padua, Italy; ^5^ Medical Oncology 2, Istituto Oncologico Veneto IRCCS, Padua, Italy; ^6^ Thoracic Oncology, Lung Unit, P. Pederzoli Hospital, Peschiera del Garda, Italy; ^7^ Medical Oncology, Azienda Sanitaria dell’Alto Adige, Bolzano, Italy; ^8^ Medical Oncology, Santa Chiara Hospital, Trento, Italy

**Keywords:** small cell lung cancer (SCLC), immune checkpoint inhibitors, immunotherapy, fragile patients, predictive factor

## Abstract

Small cell lung cancer (SCLC) represents about 13%–15% of all lung cancers. It has a particularly unfavorable prognosis and in about 70% of cases occurs in the advanced stage (extended disease). Three phase III studies tested the combination of immunotherapy (atezolizumab, durvalumab with or without tremelimumab, and pembrolizumab) with double platinum chemotherapy, with practice-changing results. However, despite the high tumor mutational load and the chronic pro-inflammatory state induced by prolonged exposure to cigarette smoke, the benefit observed with immunotherapy is very modest and most patients experience disease recurrence. Unfortunately, biological, clinical, or molecular factors that can predict this risk have not yet been identified. Thanks to these clinically meaningful steps forward, SCLC is no longer considered an “orphan” disease. Innovative treatment strategies and combinations are currently under investigation to further improve the expected prognosis of patients with SCLC. Following the recent therapeutic innovations, we have reviewed the available literature data about SCLC management, with a focus on current unmet needs and potential predictive factors. In detail, the role of radiotherapy; fragile populations, such as elderly or low-performance status patients (ECOG PS 2), usually excluded from randomized studies; predictive factors of response useful to optimize and guide therapeutic choices; and new molecular targets and future combinations have been explored and revised.

## 1 Introduction and the State of the Art

Small cell lung cancer (SCLC) accounts for about 13%–15% of all new lung cancer diagnoses. About 70% of SCLC are diagnosed at an advanced stage (1). Platinum-based chemotherapy is the standard of care for both limited disease (LD) and extensive disease (ED). Although this treatment favors survival and disease control, most patients relapse, and overall survival (OS) reaches a maximum of 2 years in 21% and 7% of LD and ED, respectively (2). However, the advent of ICIs (immune checkpoint inhibitors), including PD-1 (inhibitors of programmed cell death protein 1) and PD-L1 (programmed death-ligand 1), in the therapeutic landscape of this aggressive tumor started to change the outcome of patients with ED-SCLC.

The following review reports the state of the art, as well as recent data with immunotherapy in SCLC treatment, with a focus on unmet needs and potential predictive factors.

## 2 The Role of ICIs in SCLC Treatment

### 2.1 Biological Rationale

It has been hypothesized that genomic instability due to the expression of two defective tumor suppressor genes (*TP53* and *RB1*), thus perpetuating the generation of tumor-associated antigens (3) and the long-term exposure to smoke, thus inducing smoking signatures (4), makes SCLC one of the tumors with the highest tumor mutational burden (TMB) but low immunogenicity (SCLC has low MHC I expression levels, and its mutation products is difficult to be recognized by CD8 T-cell receptor). Furthermore, chemotherapy may induce immunogenic cell death that results in prompt release of pro-inflammatory cytokines and tumor antigens in the tumor microenvironment (TME), thus enhancing tumor immunogenicity (5). Although SCLC appears morphologically homogeneous, the latest data from murine models and human tumors indicate the existence of SCLC subtypes, classified on differential expression of these transcription factors: ASCL1, NEUROD1, POU2F3, or YAP1 with different therapeutic vulnerabilities (6). Among them, the SCLC-inflamed tumor, characterized by overexpression of immune genes such as those of the STING pathway, showed better survival with chemoimmunotherapy than other subtypes (7). However, although a phenotype characterized by high immune cell infiltration in TME showed a prognostic value in SCLC (8), it was not associated with other well-known candidate immune-biomarkers such as PD-L1 or TMB or with tumor response in patients treated with immunotherapy (9). Thus, by trying to understand the immune microenvironment, we get to know better the immunobiology of SCLC. Identifying the predictive biomarkers of response to immunotherapy in patients with SCLC and determining the strategies to overcome resistance to ICIs are future challenges.

### 2.2 Update on Treatment Options for Limited-Stage Disease

Limited-stage SCLC (LS-SCLC), meaning a tumor limited in one hemithorax and feasible radiation field, accounts for about 40% of SCLC (<5% SCLC in early stages). The role of surgery is still controversial even in early-stage SCLC, where surgery may be considered within a multimodal approach in very selected patients (10). As reported in a Cochrane systematic review published in 2017, the randomized controlled trials (RCTs) did not demonstrate a clear benefit from surgical resection in SCLC stage I–III (11). Although multiple retrospective and observational studies demonstrated the advantage of surgery for local control in the early stage I–IIA of the disease (12), the indication of surgery plus chemotherapy remains controversial. Therefore, according to the ESMO guidelines, surgery should be taken as a treatment option in patients with clinical stages I and II (cT1-2N0) and in those suspected cases with mixed SCLC histology and non-small cell lung cancer (NSCLC) (10). Otherwise, the current standard of care in SCLC of limited stage (stage I–III) consists of thoracic radiation (45 Gy in 30 fractions twice daily) plus platinum-etoposide (PE) chemotherapy (13). The advantage of this treatment is that it can be applied at full dose in patients during treatment with concomitant chemoradiotherapy (CRT) with a favorable toxicity profile. If patients are not suitable for cisplatin, carboplatin-etoposide is another treatment choice (14).

An increasing median OS was observed across CRT trials in LD-SCLC, due to technological advances and dose fractionation of radiotherapy (13). More recent trials exploring higher radiation dose schemes report even better survival outcomes, with a median OS of 37–39 months (15, 16).

However, a 70% risk of recurrence at 5 years was reported in the best-case scenarios, and maintenance or consolidation therapy strategies did not achieve a significant survival benefit (17).

Although studies are still ongoing, beneficial effects are speculated from the introduction of immunotherapy plus chemoradiation in both therapy choices, either concurrent or consolidative. The advantage of combining immunotherapy with CRT has been shown in different preclinical studies; radiotherapy used for the treatment of a primary tumor may cause the release of tumor antigens followed by a tumor-specific immune response, which is intensified by immune-stimulating elements (18). According to the abscopal effect, while radiotherapy causes a local tumor response at a targeted site, it may also cause a tumor response in non-targeted sites (metastatic disease).

We have analyzed four randomized trials, studying the concomitant therapy: immunotherapy plus CRT in LS-SCLC (**Table 1**). STIMULI (NCT02046733) is a phase II trial that studied the efficacy and tolerability of consolidation of nivolumab and ipilimumab for four cycles followed by nivolumab for 1 year versus observation after chemoradiation therapy and prophylactic cranial irradiation (PCI) in LS-SCLC (19). The statistical analysis plan considered PFS as the only primary endpoint. In total, 153 patients were randomized, and after a follow-up of 22.4 months, the trial confirmed that there were no benefits in PFS or OS with the addition of nivolumab and ipilimumab (19). Furthermore, 50% of patients included in the experimental arm were unable to receive the full course of immunotherapy due to its toxicity. However, it was outlined how biobanking will be used to investigate hematological profiles and other biomarkers to define a group of patients that may benefit from the addition of immunotherapy to standard CRT. This study began in July 2014 but was terminated early in 2019 due to slow accrual. ADRIATIC (NCT03703297) is a phase III study that evaluates the efficacy of durvalumab or durvalumab plus tremelimumab compared to placebo for consolidation in patients with LS-SCLC who have not progressed after concomitant CRT (20). PFS and OS are the primary endpoints. The study started in September 2018 and will end in May 2024. LU-005 (NCT03811002) is a phase II/III trial that studies CRT compared to atezolizumab plus CRT (21). PFS is the primary endpoint of phase II and OS is the primary endpoint of phase III. Atezolizumab is administered every 3 weeks in association with radiotherapy up to 12 months in total. The stratification variables are performance status (PS 0/1 vs. 2), sex, use of chemotherapy (cisplatin vs. carboplatin), and radiation fractionation (twice daily at 45 Gy vs. once daily at 66 Gy). PCI (25 Gy in 10 fractions) is recommended in patients with a complete or almost complete response to therapy. This study opened to accrual in May 2019 and will end in December 2026. Moreover, ACHILES (NCT03540420), a phase II randomized trial comparing atezolizumab vs. observation after concurrent CRT (primary end-point is a 2-year OS rate) (22), and the NCT04189094, a phase II trial evaluating the role of adding sintilimab, an antiPD1 antibody, to chemoradiotherapy in LD-SCLC, are still ongoing. Interestingly, current ongoing trials are evaluating the combination of anti-PD1/anti-PDL1 and anti-TIGIT. In this light, the NCT04308785 represents a phase II study concentrated on the efficacy and safety of atezolizumab associated or not with tiragolumab (anti-TIGIT) as consolidation therapy in LD-SCLC patients who have not progressed during/after CRT (23), while NCT04952597 phase II trial examines the combination of ociperlimab plus tislelizumab plus concomitant CRT.

**Table 1:** Selected randomized clinical trial testing immunotherapy in SCLC limited disease.

Trial	Ph	Setting	Study Arm(s): E) Experimental; C) Control	*N*	Primary End-point(s)	Main Results/Status	Start Date–Estimated completion rate
STIMULI (NCT02046733)	II	Maintenance after CRT	E) nivolumab + ipilimumab C) observation	E) 78 C) 75	PFS, OS	mPFS: 10.7 vs. 14.5 [HR = 1.02 (0.66-1.58), 2-sided *p* = 0.93]; mOS: NR vs. 32.1 [HR = 0.95 (0.59-1.52), *p* = 0.82]	July 28, 2014–January 2022 (completed early in 2019)
ADRIATIC (NCT03703297)	III	Maintenance after CRT	E) durvalumab +/- tremelimumab C) placebo	724	PFS, OS	Ongoing	September 27, 2018–May 10, 2024
LU-005 (NCT03811002)	II/III	Concurrent with CRT	E) CRT + atezolizumab C) CRT	506	PFS, OS	Ongoing	May 28, 2019–December 28, 2026
ACHILES (NCT03540420)	II	Maintenance after CRT	E) atezolizumab C) observation	212	2-year survival	Ongoing	July 31 2018–December 2026
NCT04189094	II	Induction and maintenance after CRT	E) sintilimab + PE → CRT → sintilimab C) PE → CRT	140	PFS	Ongoing	January 1, 2020–July 1, 2023
NCT04308785	II	Maintenance after CRT	E) atezolizumab + tiragolumab C) atezolizumab + placebo	150	PFS	Ongoing	December 1, 2021–February 15, 2025
NCT04952597	II	Concurrent and maintenance after CRT	E) CRT + ociperlimab + tislelizumab → ociperlimab + tislelizumab E) CRT + tislelizumab → tislelizumab C) CRT	120	PFS	Ongoing	July 15, 2021–March 30, 2024

PE, platinum-etoposide; CRT, concomitant chemoradiotherapy.

### 2.3 Novel Treatment Options for Extended Disease

Although current SCLC treatment remains “one size fit all”, promising results were reported in the recent phase III studies including immunotherapy, which led to regulatory drug agency approval of immuno-including regimens in the first-line setting.

#### 2.3.1 First-Line Treatment

Before the arrival of ICIs, chemotherapy with PE was considered the frontline SoC regimen for ED-SCLC for almost 30 years (24). With this regimen, ORR reached 60%–80% but responses were transient (PFS 3–6 months) and the median OS was limited (8–10 months). Recently, three phase III trials have tested the combination of ICIs (atezolizumab, durvalumab +/- tremelimumab, and pembrolizumab) with chemotherapy as first-line setting. Overall efficacy and toxicity were comparable across the studies, whereas the percentages of included patients with brain metastases or treated with PCI were different. In general, ICI introduction in the treatment landscape of SCLC represents an important and well-accepted step forward in the therapeutic strategy of ED-SCLC (**Table 2**).

**Table 2:** Selected randomized clinical trial testing immunotherapy in SCLC extended disease.

Trial	Ph	Setting	Study Arm(s)	*N*	Primary End-point (s)	Main Results	Safety(AEs Grade 34)
IMpower133 (NCT02763579)	III	1-L	E) CP/ET + atezolizumab C) CP/ET + placebo	E) 201 C) 202	OS, PFS	mOS: 12.3 vs. 10.3 [HR = 0.70 (0.54-0.91), *p* = 0.007] mPFS: 5.2 vs. 4.3 [HR = 0.77 (0.62-0.96), *p* = 0.02]	Any G3/4: 57.1% vs. 56.1%; irAE: 39.9% vs. 24.5%
CASPIAN (NCT03043872)	III	1-L	E1) PE + durvalumab E2) PE + durvalumab + tremelimumab C) PE + placebo	E1) 268 E2) 268 C) 269	OS (E1 vs. C) OS (E2 vs. C)	mOS (E1 vs. C): 12.9 vs. 10.5 [HR 0.71 (0.60-0.86), *p* = 0.0003] mOS (E2 vs. C): 10.4 vs. 10.5	Any G/4: 60% (E1) vs. 59% (C) irAE: 20% (E1) vs. 3% (C)
Keynote-604 (NCT03066778)	III	1-L	E) PE + pembrolizumab C) PE + placebo	E) 223 C) 222	PFS, OS	mPFS: 4.5 vs. 4.3 [HR 0.75 (0.61-0.91), *p* = 0.0023] mOS: 10.8 vs. 9.7 [HR 0.80 (0.64-0.98), *p* = 0.0164]	Any G3/4: 76.7% vs. 74.9%; irAE: 24.7% vs. 10.3%
REACTION (NCT02580994)	II	1-L^*^	E) PE + pembrolizumab C) PE	E) 58 C) 61	PFS	mPFS: 4.7 vs. 5.4 [HR 0.84 (0.65-1.09), *p* = 0.194]	Any G3/4: 41.7% vs. 34.4%
NCT02359019	II single arm	Maintenance	Pembrolizumab for 2 years	45	PFS	mPFS: 1.4 12-month PFS: 13%	The only G3 ≥5% was hyponatremia
Checkmate 451 (NCT02538666)	III	Maintenance	E1) ipilimumab + nivolumab → nivolumab E2) nivolumab C) placebo	E1) 278 E2) 279 C) 273	OS (E1 vs. C)	mOS: 9.2 vs. 9.6 [HR 0.92 (0.75-1.12), *p* = 0.37]	Any G3/4: 52.2% vs. 8.4%
Keynote-028 (NCT02054806) Keynote-158 (NCT02628067)	Ib II	2-L and beyond	Pembrolizumab	107	ORR	ORR: 19.3% (11.4-29.4)	Any G3/4: 9.6%
Checkmate 032 (NCT01928394)	I/II	2-L and beyond	E) nivolumab + ipilimumab → nivolumab C) nivolumab	E) 96 C) 147	ORR	ORR: 21.9% vs. 11.6%	Any G3/4: 37.5% vs. 12.9%
Checkmate 331 (NCT02481830)	III	2-L	E) nivolumab C) topotecan or amrubicin	E) 284 C) 285	OS	mOS: 7.5 vs. 8.4 [HR 0.86 (0.72-1.04), *p* = 0.11]	Any G3/4: 13.8% vs. 73.2%
NCT01693562	I/II	2-L and beyond	Durvalumab for 12 months	21	Safety	ORR: 9.5% mPFS: 1.5 months mOS: 4.8 months	No G3/4
BALTIC (NCT02937818)	II	2-L	Durvalumab + tremelimumab	21	ORR	ORR: 9.5%	Any G3/4: 48%

CP, carboplatin; ET, etoposide; PE, platinum-etoposide; irAEs, Immune-related adverse events.

^*^Patients with an objective response after two cycles of induction chemotherapy with 2 cycles.

The IMpower133 trial evaluated the efficacy of adding the PD-L1 inhibitor atezolizumab to the standard carboplatin-etoposide in 403 naive patients with ED-SCLC, considering as stratifying factors sex, ECOG PS, and the presence of brain metastases (25, 26). After four cycles of treatment, PCI was included during the atezolizumab/placebo maintenance period; meanwhile, consolidation thoracic radiation was not considered. Primary endpoints were reached, with an important reduction of 30% and 23% risk of death and progression, respectively, in patients treated with atezolizumab. Median OS was 12.3 months and 10.3 months, respectively, for experimental and placebo arm (HR: 0.70; 95% CI: 0.54–0.91, *p* = 0.007); PFS was 5.2 months and 4.3 months, respectively, for atezolizumab and control arm (HR: 0.77; 95% CI: 0.62–0.96, *p* = 0.02) (26). One-year OS rate was 51.7% and 38.2% in patients undergoing chemotherapy plus atezolizumab vs. chemotherapy alone, respectively, regardless of PD-L1 expression and blood tumor molecular burden. According to these results, the combination of carboplatin, etoposide, and atezolizumab is considered as the new standard treatment for ED-SCLC in the first-line setting.

The CASPIAN trial tested the efficacy of the PD-L1 inhibitor durvalumab +/- CTLA-4 inhibitor tremelimumab, in combination with standard PE in 805 ED-SCLC naïve patients (27, 28). The control group was represented by PE alone for up to six cycles. In this trial, PCI was allowed in the control arm following chemotherapy at the investigator’s discretion, but it was not allowed in the immunotherapy groups before discontinuation of all study treatments. The co-primary endpoint was OS for durvalumab platinum-etoposide compared to chemotherapy, and for durvalumab/tremelimumab plus platinum-etoposide compared to chemotherapy. At the updated median follow-up after >3 years, combining durvalumab with platinum-etoposide significantly improved OS over only chemotherapy (12.9 vs. 10.5 months; HR: 0.71; 95% CI: 0.60–0.86; *p* = 0.0003) (27). Although the combination of durvalumab/tremelimumab plus PE numerically improved OS vs. PE, it was not statistically significant. In consideration of these results, durvalumab plus cisplatin or carboplatin etoposide has been approved by the Food and Drug Administration (FDA) and the European Medicine Agency (EMA) as first-line treatment in patients with ED-SCLC.

The KEYNOTE 604 trial investigated the efficacy of the PD-1 inhibitor pembrolizumab plus PE vs. chemotherapy alone in 453 ED-SCLC naive patients (29). One of its primary endpoints showed an important PFS improvement by adding pembrolizumab to chemotherapy (HR: 0.75; 95% CI: 0.61–0.91), also a prolonged OS (10.8 vs. 9.7 months); the pre-specified significance threshold was not reached (HR: 0.80; 95% CI: 0.64–0.98; *p* = 0.0164).

More recently, the REACTION trial randomized patients, with a response after two cycles of chemotherapy, to be treated with pembrolizumab in combination with chemotherapy or chemotherapy alone (30). The primary PFS endpoint was not reached (4.7 vs. 5.4 months, HR: 0.84; 80% CI: 0.65–1.09, *p* = 0.194). However, a statistically significant OS improvement (12.3 vs. 10.4 months, HR 0.73; 80% CI: 0.54–1.00) was reported.

Overall, a grade 3 or higher toxicity rate was observed during immune-chemotherapy combinations compared to chemotherapy alone, although an expected increase in immune-related AEs was reported in the experimental arms. However, data on long-term or deterioration in quality of life are limited.

#### 2.3.2 Maintenance Therapy With ICIs

The efficacy of ICIs as a maintenance strategy in ED-SCLC is still controversial. In a phase two study, 8 weeks after the last cycle of PE chemotherapy, pembrolizumab was started as maintenance therapy for up to 2 years (31). Although both median PFS and OS were not significantly improved by pembrolizumab, the 1-year PFS and OS rate were 13% and 37%, respectively, showing that a subgroup of patients could have a clinical benefit. However, none of the biomarkers analyzed, including PD-L1, were predictive of a better response to immunotherapy.

As expected by previous studies with anti-CTLA4 ipilimumab (32) in combination with chemotherapy in ED-SCLC, no significant benefit was reported in a phase III study of immunotherapy doublet with the anti-PD-L1, nivolumab, and the anti-CTLA4, ipilimumab, as maintenance therapy for ED-SCLC (33). A total of 834 patients enrolled in the Checkmate 451 trial did not progress after receiving four cycles of platinum-based chemotherapy. These patients were randomized to receive immunotherapy with nivolumab and ipilimumab, nivolumab alone, or placebo for 2 years. The OS did not improve with nivolumab plus ipilimumab vs. placebo (HR: 0.92; 95% CI: 0.75–1.12; *p* = 0.3693) or with nivolumab vs. placebo (HR: 0.84; 95% CI: 0.69–1.02), but still there was a modest improvement in PFS with ipilimumab plus nivolumab (HR: 0.72; 95% CI: 0.60–0.87) and nivolumab (HR: 0.67; 95% CI: 0.56–0.81) compared to placebo.

Finally, the addition of an anti-PD-L1 therapy (atezolizumab or durvalumab) to the standard platinum-etoposide chemotherapy, and then keeping immunotherapy as maintenance, improved both PFS and OS (25, 28). On the other hand, the use of an anti-PD1 therapy (pembrolizumab) for the same purpose showed a similar benefit that was instead statistically significant only for PFS (29). According to previous studies in other tumors (32), although the overall benefit is only about 2 months in the extension of median survival, there are potential advantages for long-term survivors, looking at the tail of survival curves. In fact, the 2-year survival rate increased from 11% to 22%, suggesting that some patients with SCLC have a significant benefit with immunotherapy, but useful biomarkers for their *a priori* identification are still lacking.

#### 2.3.3 Second-Line Treatment and Beyond

Unfortunately, most patients relapse within 6 months after first-line chemotherapy. The second-line treatment response rates depend on the treatment-free interval (TFI) and are approximately 20%–30% in platinum-sensitive patients (TFI ≥3 months) and 15% in platinum-resistant patients (TFI <3 months). According to clinical guidelines, the two possible second-line options for patients with ED-SCLC who progressed after platinum-based first-line chemotherapy are the topoisomerase I inhibitor topotecan and anthracycline-based regimes, including cyclophosphamide plus doxorubicin and vincristine (CAV) (10). The latest option was commonly used before a randomized trial with topotecan vs. CAV, which showed similar outcomes in both treatment arms, but intravenous topotecan showed better tolerability (34), resulting in the preferred standard of care nowadays. However, in platinum-sensitive patients, a rechallenge with PE should be also considered as a reasonable second-line option. In fact, a phase III trial recently showed that carboplatin plus etoposide had a significant improvement in PFS compared to topotecan (4.7 vs. 2.7 months, HR: 0.57; 95% CI 0.41–0.73; *p* = 0.0041), with a similar safety profile (35). In the ESMO therapeutic algorithm, lurbinectidin (selective inhibitor of RNA polymerase II) was also introduced as an alternative option for recurrent SCLC (10). This drug was recently approved by the FDA, according to the results of a phase II single-arm trial (NCT02454972), in which the single-agent lurbinectedin showed significant activity as second-line therapy. Overall, patients reported an ORR equal to 35.2% (22.2% in platinum-resistant and 45% in platinum-sensitive patients), a median duration of response up to 5.3 months (36), and a median OS of 9.3 months, with a manageable safety profile (37). Meanwhile, the combination of lurbinectedin plus doxorubicin explored in the phase III trial ATLANTIS (NCT02566993) vs. investigator’s treatment choice (topotecan or CAV: cyclophosphamide, doxorubicin, vincristine) did not improve the prespecified endpoint of OS (38).

Promising preliminary antitumor activity and a good safety profile were shown with ICIs in patients progressed after standard first-line chemotherapy. The administration of pembrolizumab 200 mg every 3 weeks, as the standard dose, was tested in a phase Ib (Keynote-028) and a phase II (Keynote-158) trial in different tumor types, including SCLC. Overall, an ORR (primary endpoint) of 19.3% was reported regardless of PD-L1 expression. On this basis, in June 2019, the FDA accelerated the approval of pembrolizumab for the treatment of metastatic SCLC patients with disease progression after receiving platinum-based chemotherapy and at least one other prior line of therapy.

The use of nivolumab in SCLC pretreated patients has been evaluated in the phase I/II Checkmate 032 (39) and in the phase III Checkmate 331 (40) clinical trials. The first one is a basket trial that studied the activity of nivolumab alone and nivolumab plus ipilimumab in different tumors including metastatic SCLC. Overall, an objective response of 10% was observed in patients treated with nivolumab alone, 23% in those treated with nivolumab 1 mg/kg plus ipilimumab 3 mg/kg, and 19% in those treated with nivolumab 3 mg/kg plus ipilimumab 1 mg/kg. Like pembrolizumab studies, the response rate was not related to PD-L1 status. Further analysis after 18 months of follow-up showed an ORR of 11% in patients treated with nivolumab alone and of 25% in patients treated with nivolumab plus ipilimumab (41). These early results, in August 2018, led the FDA to accelerate the approval of nivolumab for pretreated SCLC patients. Recently, results of the expansion cohort of patients randomized to nivolumab vs. nivolumab plus ipilimumab were published, reporting an ORR of 11.6% in the group with nivolumab alone, and 21.9% in the combination group that experienced more frequent G3–G4 adverse events (12.9% and 37.5% in the nivolumab and nivolumab + ipilimumab group, respectively) and four deaths due to toxicity (autoimmune-related hepatitis, pneumonitis and encephalitis, and autoimmune colitis).

The second study, Checkmate 331, compared nivolumab vs. chemotherapy with topotecan or amrubicin as second-line treatment (42). Patients were grouped as platinum responders and non-responders. Although this trial did not reach its primary endpoint [median OS was 7.5 vs. 8.4 months in the nivolumab and chemotherapy arm (HR: 0.86; CI: 95%: 0.72–1.04)], the HR for OS in patients who did not respond to cisplatin was 0.71 (95% CI: 0.54–0.94). Additionally, the nivolumab group reported 55% of all grade AE vs. 90% in the chemotherapy arm (40).

Durvalumab was reported to have similar results, received every 2 weeks at a dose of 10 mg/kg, in a phase I/II study that included 21 patients with pretreated ES-SCLC disease (43). Patients were treated for up to 1 year and reported a median OS of 4.8 months, PFS of 1.5 months, and a 1-year OS rate of 27.6%. In addition, an ORR of 9.5% was recently observed with durvalumab + tremelimumab in preliminary analysis of the phase II BALTIC study (NCT02937818) (44).

The anti-PD-L1 atezolizumab as single therapy did not show significant results in pretreated patients vs. topotecan (up to six cycles) or re-induction chemotherapy in the randomized phase II IFCT-1603 study, which included 73 patients with ES-SCLC disease after failure of first-line PE-basing chemotherapy (45).

Overall, the potential use of ICIs in the second-line setting still requires further evidence. Furthermore, considering that immunotherapy is currently included in the first-line standard of care approach, it must be taken into consideration the lack of data on the role of ICIs rechallenge in patients whose disease has progressed after first-line immune-based treatment.

## 3 Open Issues

### 3.1 Radiotherapy: The Role of Consolidation Treatment and Prophylactic Cranial Irradiation

Thoracic radiotherapy (TRT) combined with chemotherapy is the standard treatment in patients with limited disease. In ED-SCLC, the importance of consolidation of TRT in patients with a good response to first-line treatment has become increasingly recognized (46). The ASTRO guidelines conditionally recommend thoracic radiotherapy to 30 Gy in 10 fractions within 6 to 8 weeks of chemotherapy completion and before maintenance immunotherapy, in patients with ED-SCLC who respond to chemotherapy and immunotherapy, and in case of residual disease in the thorax. At the ASCO 2021, SBRT was suggested to be applied more frequently in early-stage SCLC patients not eligible for resection, or who refuse surgery. Additionally, retrospective data suggest that this strategy is likely safe and effective. Therefore, ASTRO guidelines have recently incorporated SBRT as an acceptable treatment option for early-stage, node-negative, and medically inoperable SCLC.

Prophylactic cranial irradiation (PCI) is still controversial, following the publication of a Japanese randomized phase III trial that found that magnetic resonance imaging (MRI) surveillance could replace PCI for extensive-stage disease (47). In this trial, patients with ED-SCLC who responded to platinum-based doublet chemotherapy and with no brain metastases on MRI were randomly assigned (1:1) to receive PCI (25 Gy in 10 daily fractions of 2.5 Gy) or observation. The primary endpoint was OS. All patients underwent brain MRI every 3 months in a 12-month period followed by another brain MRI at 18 and 24 months after enrolment. The study showed that there was no improvement in OS with PCI therapy compared to observation (11.6 vs. 13.7 months, HR = 1.27, 95% CI 0.96–1.68, *p* = 0.094), concluding that PCI is not essential for ED-SCLC responders to initial chemotherapy, without evidence of brain metastases (48).

However, due to the incidence of brain metastases at diagnosis (about 18% of cases of ED-SCLC, which increase to 80% at 2 years), PCI is still recommended in patients who respond to treatment in both LD and ED-SCLC (49). However, active surveillance with a brain MRI every 12 weeks seems to be an acceptable option, especially to preserve patients’ quality of life (48).

Although consistent data are not available for SCLC in the immunotherapy era, the safety and efficacy data obtained in NSCLC about the integration of ICIs and radiotherapy may support the feasibility of this approach. In this light, it will be crucial to better define the potential (positive and negative) synergy between local and systemic therapy in both LD and ED-SCLC, similar to what is recognized in stage III NSCLC and in the oligometastatic/oligoprogressive setting. The patient’s condition, stage, and characteristics of the disease, response to therapy, dosage and schedule of TRT/PCI, as well as the future availability of new drugs or combinations may influence the decision-making process in SCLC. In conclusion, the integration of thoracic radiotherapy in patients with ED-SCLC who after chemotherapy have persistent intrathoracic disease, as well as the role of PCI and immunotherapy in the metastatic setting, remains an important unanswered question, prioritizing the need for *ad hoc* trials.

### 3.2 Frail Population

Although etoposide plus carboplatin was accepted as a tolerable and equivalent regimen in terms of efficacy compared to etoposide and cisplatin, a review of the Alberta Cancer Registry showed that 32% of elderly patients (age 75+) were not treated with chemotherapy (50). Moreover, the randomized phase III trials with ICIs enrolled patients with a median age ≤70 years; they included only patients with good performance status (PS, 0-1) (23, 25, 27). In contrast, in real life, there are more and more cases of elderly patients with median age ≥70 years (51). Overall, 52% of the patients treated with chemotherapy completed all cycles and 34% of them underwent at least one dose reduction. Patients who completed all cycles with a dose reduction had a lower risk of death of 1.02 (95% CI: 0.57–1.82) compared to a risk of death of 2.72 (95% CI: 1.52–4.87) for patients who did not complete therapy. Furthermore, phase II studies aimed to point out that carboplatin and etoposide dose modifications in the elderly reported similar survival benefits *versus* standard doses (52). Therefore, elderly patients should receive standard treatment, but they may also require dose modifications.

Recently, NSCLC studies showed that immunosenescence, defined as the gradual deterioration of the immune system caused by natural advances in age, seems to be related to decreased efficacy of ICIs, regardless of the age (53). However, survival data from phase III clinical trials with ICIs are controversial in SCLC elderly population. In the KEYNOTE 604, similar magnitudes of survival benefit were reported with the use of pembrolizumab despite the patient’s age (29). In contrast, in the CASPIAN trial, durvalumab was significantly effective in patients aged <65 years [HR 0.72, (95% CI: 0.56–0.92)] but not in patients aged ≥65 years [HR 0.84 (95% CI: 0.62-1.12)] (27), whereas the anti-PD-L1, atezolizumab, in the IMpower133 trial seemed to be more effective in patients aged ≥65 years [HR 0.53 (95% CI: 0.36–0.77)] than patients aged <65 years [HR 0.92 (95%: 0.64–1.32)]

In real-world data, up to 60% of elderly patients have a PS equal to 2, resulting in worse survival (54). Furthermore, they were generally also affected by at least two chronic comorbidities, followed by a higher probability of exposure to polypharmacy, which can affect the efficacy of ICIs (55). In this scenario, the REACTION trial hypothesized an interesting strategy. In this phase II trial, there were randomized patients with complete or partial response after two cycles of PE induction. In this study, 5% of the patients enrolled had an ECOG PS 2, but those patients who upgraded to PS 1 or 0 with treatment benefit were eligible for the immune-chemotherapy strategy (30). Finally, a large sample of PS 2 patients will be enrolled in the ongoing phase II SPACE trial (NCT04221529) (56) and in the phase III MAURIS trial (NCT04028050) (23), which may help to clarify whether these patients benefit or not from the addition of ICI to chemotherapy.

In terms of brain radiotherapy, the role of PCI in elderly patients is controversial. Indeed, even if PCI improved the OS in patients aged ≥70 years, it was not significantly effective among patients aged ≥80 years with SCLC (57). This finding suggests that in this group of patients, a shared decision process is necessary rather than proposing an overtreatment. Less is known in patients with ECOG-PS = 2 and those with a history of neurological conditions, such as stroke or epilepsy. Retrospective analyses have shown that PCI improves survival compared to no PCI, but its correlation with increased neurocognitive dysfunction has limited its use (57–59). In fact, a comparison of the results of cognitive tests in two RTOG trials that evaluated PCI in patients with LS-SCLC showed higher rates of cognitive decline with advanced age (60). Modern radiation techniques, hippocampal sparing, and memantine may minimize the occurrence of cognitive decline.

Geriatric Assessment Tools Geriatric oncology addresses the right approach to the care of this category of patients through the development of geriatric assessment tools to help define risks and benefits. Investigators and treating physicians are encouraged to include these tools in their clinical trials and daily practice (46). In general, clinical trials and trials that address the unique needs of the elderly are strongly recommended.

### 3.3 Potential Predictive Factors

SCLC is a highly aggressive tumor with still very poor prognosis marked by a very high proliferative rate and an early spread of metastasis. Moreover, despite the promising results, the magnitude of benefit with ICIs in SCLC is different from what was reported in NSCLC. Although SCLC has a high TMB, its immunosuppressive pattern in the stroma, the lack of antigen presentation, and the low expression of PD-L1 suggest a less immunogenic T-cell profile in SCLC compared to NSCLC (36, 39, 61). Similarly, a multiplexed quantitative immunofluorescence analysis in SCLC samples showed significantly lower levels of all TIL markers, MHC class II expression, and CD8+ T cells compared to NSCLC (62). However, high immune activity was reported in patients with SCLC and paraneoplastic syndromes, resulting in a better prognosis compared to patients without these syndromes (63).

Moreover, a clear therapeutic algorithm and consolidated data in special populations, like the elderly or patients with an ECOG PS ≥ 2, are still unavailable. Therefore, the identification of potential predictive factors of response to better guide the physician’s choice is awaited.

#### 3.3.1 Molecular Factors

##### 3.3.1.1 Gene Expression Profile

The genomic profile of SCLC shows extensive chromosomal rearrangements and a high TMB. Moreover, the dual inactivation of the tumor suppressors *TP53* and *RB1* is found in most cases with SCLC (3). Sequencing analysis on both DNA and RNA of larger cohorts of primary tumors as well as CTC-derived xenograft models confirmed this result. Furthermore, the amplification of genes from the *MYC* family (*MYC*, *MYCL*, and *MYCN*), *FGFR1* (encoding fibroblast growth factor receptor 1), and *GNAS* (encoding the α-subunit of the heterotrimeric G protein Gs) was also well described (64). Moreover, alterations in the PTEN pathway and overexpression of BCL-2 could interfere with the promotion of cell growth, proliferation, and survival in SCLC. Relapsed tumors are more frequently characterized by WNT pathway alterations, thus supposing a role for WNT signaling in chemo-resistant SCLC (65), and the heterogeneity of SCLC tumors may explain an important mechanism by which SCLC tumors evade treatment; additionally, heterogeneity itself is increased in response to treatment (66). The lineage plasticity of SCLC cells could be explained by the high levels of the stem cell transcription factor SOX2 downstream of *p53* and *RB* loss, or as a consequence of genomic amplification. Moreover, mutations in chromatin modifiers are frequent in SCLC, suggesting that alterations in epigenetic regulation may also contribute to cell fate changes. However, better understanding the TME and the molecular mechanisms underlying SCLC tumorigenesis, progression, metastasis, and response to treatment is still a challenge. Recently, some researchers have developed the first comprehensive framework to classify SCLC into four subtypes based on gene expression (6). This classification depends on the relative expression of dominant transcriptional regulators and on the substantial intra-tumoral heterogeneity that could explain the main aspects of tumor evolution, metastasis, and acquired therapeutic resistance, as well as potential targeted therapeutic strategies (64).

The first three groups are characterized by activation of the *ASCL1* (SCLC-A), *NEUROD1* (SCLC-N), and *POU2F3* (SCLC-P) genes, while the SCLC-I subtype is characterized by an inflamed gene signature with high expression of multiple immune genes, including significantly higher levels of genes indicating the presence of CD8-positive cytotoxic T cells (7). The research team first identified the four groups by applying non-negative matrix factorization to 81 SCLC patients with surgically resected tumors. The data from 276 SCLC patients enrolled in the phase III IMpower133 clinical trial were then analyzed to validate the four subtypes in the advanced stage. This study showed that SCLC-I was the most sensitive to immune checkpoint blockade, SCLC-A was the most sensitive to BCL2 inhibitors, SCLC-N was the most sensitive to Aurora kinase inhibitors (overall, more effective in those SCLC with increased MYCL expression) (67), and SCLC-P was the most sensitive to PARP inhibitors, thus suggesting different classes of drugs for different specific subtypes. This study described the subtype “*switching*” to resistance in a series of patient-derived SCLC models. Data from a mouse model also suggest that SCLC-A tends to switch to SCLC-I after being treated with chemotherapy, which could be correlated with resistance to treatment (7). Since SCLC is about 15 years behind NSCLC, in terms of developments in the field of biomarkers and personalized therapies, this emerging molecular classification represents the first step in a better understanding of the molecular pathway involved in SCLC and the choice of the best drugs for each patient, thus moving towards personalized approaches for the cure of the rare and aggressive SCLC tumor.

##### 3.3.1.2 Liquid Biopsy

A pressing issue in the SCLC field has been the small quantity of material to be used for histological diagnosis and subsequent research. Therefore, isolating circulating tumor cells (CTCs) from the blood of SCLC patients could overcome this problem (68). However, we are still far from adequate clinical trials that concentrate on tumor material collection to identify key genetic drivers of SCLC, and while liquid biopsies may represent an important factor for exploring ICI-resistance mechanisms in SCLC, this technique itself needs more evaluation.

#### 3.3.2 Immunological Factors

Exploratory biomarker analysis of principal phase III clinical trials showed that PD-L1 expression is not correlated to immunotherapy benefit in SCLC patients. The importance of TMB is more controversial, which seems to be predictive of nivolumab-ipilimumab benefit as the Checkmate-032 analysis suggests (69) but not predictive of atezolizumab benefit in the IMPOWER133 blood-based analysis (25). Similarly, in the CASPIAN trial, durvalumab plus chemotherapy resulted in improved OS compared to chemotherapy alone regardless of PD-L1 and TMB expression (28). Also, in the KEYNOTE 604 trial, both PFS and OS improved with the addition of pembrolizumab to chemotherapy, regardless of the combined positive PD-L1 expression score (29). Therefore, we cannot consider PD-L1 or TMB to be good predictive factors of response to immunotherapy in SCLC, at least so far.

#### 3.3.3 Clinical Factors

According to their different microenvironments, brain metastasis and liver metastasis deserve to be mentioned as potential predictive factors of response to immune-based chemotherapy.

Indeed, the brain metastases showed an active immune microenvironment with a PD-L1 expression of 75% in SCLC samples. However, the percentage of patients with baseline brain metastases included in phase II/III clinical trials ranged from 9% to 14.2% in the immunochemotherapy arms (25, 27, 29, 30). Moreover, all trials, except the CASPIAN trial, included only asymptomatic and treated brain metastases. Thus, the limited sample size and the limited benefit in survival by adding immunotherapy to chemotherapy do not allow conclusive results. The presence of liver metastases should be considered a negative predictive factor. In particular, in the three phase III trials, anti-PD-L1 addiction to chemotherapy did not improve survival results compared to chemotherapy alone. Accordingly, in NSCLC, the occurrence of liver metastases was associated with an immune-suppressive phenotype characterized by fewer infiltrating CD8+ T-cell densities at the invasive margin in distant tumors (66) and limited immunotherapy efficacy by macrophage-mediated elimination of T cells (70). These data support the hypothesis that there is a lack of a synergistic effect of immunochemotherapy in SCLC patients affected by liver metastases.

## 4 New Targets and Future Perspectives

Despite the high potential immunogenicity of SCLC, the magnitude of benefit with ICIs in SCLC is not the same as that reported in NSCLC patients. Different immunophenotypes, as well as the TMEs of SCLC compared with NSCLC, may explain the different efficacy of ICIs in these two diseases (61).

Recently, other immunotherapeutic approaches, used alone or in combination with ICIs, are being explored to improve the immune response in SCLC patients. These include chimeric antigen receptor (CAR) T-cell therapy, bispecific T-cell engagers (BiTEs), antibody–drug conjugates (ADC), and immunomodulators. Multiple cell surface molecules, including CD56, CD47, and delta-like ligand 3 (DLL3), have an important expression in SCLC, thus emerging as potential therapeutic targets of CART therapy (71–73) (**Table 3**).

**Table 3:** Selected clinical studies including novel drugs/novel combinations in SCLC.

Trial	Ph	Setting	Type of approach	Study Arm(s): E) Experimental; C) Control	Primary End-point(s)	Main Results/Status	Start Date–Estimated completion rate
NCT03392064	I	Relapse/Refractory SCLC	DLL3-directed CART cell therapy (AMG 119)	Single arm	DLTs	Suspended*	September 10, 2018–January 13, 2026
NCT03319940	I	Relapse/Refractory SCLC	DLL3-targeted BITEs (AMG 757)	Arm A) AMG 757 Arm C) AMG 757 with Pembrolizumab Arm D) AMG 757 with additional CRS mitigation strategies Arm E-F-G) AMG 757 with different timing of administration/schedules	DLTs	Recruiting [Results from updated analysis: DCR: 43% mDOR: 6.2 mo TRAEs G3: 25% TRAEs G4: 6%]	December 26, 2017–September 12, 2024
TRINITY (NCT02674568)	II	Relapse/Refractory SCLC [third line or later]	DLL3-targeted ADC (Rovalpituzumab Tesirine)	Single arm	ORR, OS	ORR: 12.4% (all population) 14.3% (DLL3-high population); mOS: 5.6 mo (all population) 5.7 mo (DLL3-high population); AEs G3-5: 63% [fatigue, photosensitivity reaction, pleural effusion]	January 25, 2016–October 19, 2018
TAHOE (NCT03061812)	III	Relapse/Refractory SCLC [second line; high DLL3 expression]	DLL3-targeted ADC (Rovalpituzumab Tesirine)	E) Rovalpituzumab tesirine C) Topotecan	OS	mOS: 6.3 mo vs. 8.6 mo [HR = 1.46 (1.17-1.82), *p* = 0.0051]; mPFS: 3.0 mo vs. 4.3 mo [HR = 1.51 (1.22-1.87)]; ORR: 15% vs. 21%; AEs G3-5: 56% [malignant neoplasm progression, pleural effusion, peripheral edema] vs. 57%	April 11, 2017–February 12, 2020
MERU (NCT03033511)	III	Maintenance therapy after first-line platinum-based CT	DLL3-targeted ADC (Rovalpituzumab Tesirine)	E) Rovalpituzumab tesirine C) Placebo	OS in DLL3 high population, PFS by CRAC	mOS: 8.5 mo vs. 9.8 mo [HR = 1.07 (0.84-1.36), *p* = 0.537]; PFS evaluation by CRAC not concluded due to lack of OS benefit; AEs G3-5: 59% [pleural effusion, fatigue, peripheral edema] vs. 30%	February 7, 2017–November 20, 2019
IMPULSE (NCT02200081)	II	Maintenance therapy after first line platinum-based CT	TLR 9 agonist (Lefitolimod/MGN1703)	E) Lefitolimod/MGN1703 C) Control	OS	mOS: 279 vs. 272 days [HR = 1.14 (0.73-1.76), *p* = 0.98]**; mPFS: 90 vs. 111 days [HR not determined, *p* = 0.52]	March 2014–October 5, 2017
SKYSCRAPER-02 (NCT04256421)	III	First-line ED-SCLC	Anti-TIGIT (Tiragolumab) plus anti-PDL1 agent (Atezolizumab)	E) Tiragolumab + Atezolizumab + PE C) Placebo + Atezolizumab + PE	PFS, OS	Active, not recruiting	February 4, 2020–March 21, 2024
NCT03041311	II	First-line ED-SCLC	CDK 4/6 inhibitor (Trilaciclib/G1T28) plus anti-PDL1 agent (Atezolizumab)	E) Trilaciclib + Atezolizumab + PE C) Placebo + Atezolizumab + PE	Potential to reduce CT-induced myelosuppression	Active, not recruiting	April 7, 2017–June 2021
NCT02484404	I/II	Relapse/Refractory SCLC	PARP inhibitor (Olaparib) plus anti-PDL1 agent (Durvalumab)	Single arm	ORR	ORR: 10.4%; mPFS: 1.8 mo; mOS: 4.1 mo	June 20, 2015–January 30, 2023
NCT04728230	I/II	First-line ED-SCLC	PARP inhibitor (Olaparib) plus anti-PDL1 agent (Durvalumab)	Single arm (+ chemotherapy and radiotherapy)	DLTs	Recruiting	January 5, 2021–July 01, 2022
MK 7339-013/KEYLYNK-013 (NCT04624204)	III	LD-SCLC	PARP inhibitor (Olaparib) plus anti-PD1 agent (Pembrolizumab)	E) Pembrolizumab + PE (4 cycles) with CRT → pembrolizumab (9 cycles) E) Pembrolizumab + PE (4 cycles) with CRT → pembrolizumab (9 cycles) + olaparib C) PE (4 cycles) with CRT → placebo	PFS, OS	Recruiting	December 8, 2020–October 28, 2027
ALTER-1202 (NCT03059797)	II	Relapse/Refractory SCLC [third line]	Multikinase antiangiogenetic agent (Anlotinib)	E) Anlotinib C) Placebo	PFS	mPFS: 4.1 mo vs. 0.7 mo [HR = 0.19 (0.12-0.32), *p* < 0.0001]; mOS: 7.3 mo vs. 4.9 mo [HR = 0.53 (0.34-0.81), *p* = 0.0029]; AEs G3-4: 51.9% [hypertension, hand foot syndrome] vs. 43.6%	March 27, 2017–May 6, 2019
PASSION (NCT03417895)	II	Relapse/Refractory SCLC [second line]	Multikinase antiangiogenetic agent (Anlotinib) plus novel antiPD1 (Camrelizumab)	Arm A) Camrelizumab + Apatinib Arm B) Camrelizumab + Apatinib (5 days on, 2 days off) Arm C) Camrelizumab + Apatinib (7 days on, 7 days off)	ORR	ORR: 34% [ORR in chemosensitive pts 37.5%; ORR in chemoresistant pts 32.3%]; mPFS: 3.6 mo; mOS: 8.4 mo; AEs 3-4: 72.9%	February 5, 2018–March 2020

SCLC, small cell lung cancer; DLL3, delta-like ligand 3; CART, chimeric antigen receptor T cells; DLTs, dose-limiting toxicities; BITEs, bispecific T-cell engagers; CRS, cytokine release syndrome; DCR, disease control rate; mDOR, median duration of response; mo, months; ADC, antibody–drug conjugates; ORR, objective response rate; mOS, median overall survival; HR, hazard ratio; AEs, adverse events; PFS, progression-free survival; CT, chemotherapy; PE, platinum-etoposide; CRT, CRT, concomitant chemoradiotherapy; CRAC, Central Radiographic Assessment Committee; TLR, toll-like receptor; PE, platinum-etoposide; CDK 4/6, cyclin-dependent kinase 4/6; PARP, poly (ADP-ribose) polymerase; RT, radiotherapy; PTS, patients.

*Study on enrolment hold, may potentially resume. No active subjects on trial.

**Benefit in OS was seen in patients with a low frequency of activated CD86+ B cells [HR = 0.53, (0.26–1.08)] and in patients with chronic obstructive pulmonary disease (COPD) [HR = 0.48 (0.20–1.17)].

T cell-based therapy is an MHC-independent therapeutic option, where chimeric antigen receptors are recombinant receptors for tumor-specific antigens, engineered into T cells to allow expression, expansion, and antitumor specificity (74).

AMG 119, a DLL3-directed CART cell therapy, showed a potent antitumor response in preclinical models (75) and is being studied in an ongoing phase I trial that includes patients with advanced SCLC in progression after receiving at least one platinum-based regimen (NCT03392064). Unlike CART, BiTEs are recombinant bispecific proteins that simultaneously target a T-cell surface molecule (such as CD3) and a tumor-specific surface antigen, facilitating both T-cell adherence and antitumor response independent of MHC (76).

Preclinical studies showed that the DLL3-targeted BITEs AMG 757 demonstrated a potent and specific killing activity in SCLC cell lines as well as orthotopic and patient-derived xenograft (PDX) mouse models with DLL3 expression, by inducing T-cell activation and its redirection against tumor cells (77). AMG 757 is currently being evaluated alone or in combination with pembrolizumab in a phase I trial (NCT03319940) (78). In the updated analysis of 10 cohorts including 64 patients, AMG757 at doses up to 100 mg reported promising results in terms of response rate (43% of the disease control rate, with 13% of PR) and median response duration (6.2 months), with a relative safety profile (grade ≥3 and 4); treatment-related adverse events (AEs) occurred in 25% and 6% of cases, respectively. Cytokine release syndrome occurred in 42% of patients, mainly as mild grade toxicity (79).

Rovalpituzumab tesirine (Rova-T), a DLL3-targeted ADC, has been largely investigated in different settings of SCLC, first of all in the third-line (phase II single-arm TRINITY) (80), then in the second-line (phase III TAHOE) (81), and later as first-line maintenance therapy after platinum-based chemotherapy (phase III MERU) (82). Unfortunately, it does not show the expected activity, thus failing to improve the landscape of SCLC treatment.

Vaccines, such as fucosyl GM-1, GD3 ganglioside, polysialic acid, and dendritic cell-based p53, are also a potentially promising strategy in the management of SCLC but remaining under investigation (83).

Lefitolimod, a toll-like receptor (TLR) 9 agonist, is an immunomodulator drug studied as maintenance therapy after first-line chemotherapy in the phase II trial IMPULSE (84). Although this trial did not demonstrate an OS benefit in the intention-to-treat population, a subgroup analysis of patients with a low frequency of activated CD86+ B cells resulted in a potential OS benefit.

Promising activity in SCLC is being shown with the combination of ICIs and anti-LAG-3 (78) as well as with anti-TIM-3 agents (85), which are both correlated with the development of resistance to PD-1 blockade (80). Similarly, SKYSCRAPER-02 (NCT04256421) is a phase III randomized, double-blind, placebo-controlled trial investigating the addition of another ICI, tiragolumab (anti-TIGIT agent), to first-line atezolizumab, carboplatin, plus etoposide in patients with ES-SCLC.

Given the high expression levels of DNA damage response (DDR) proteins, such as PARP, ATR, CHK1, and WEE1 in SCLC, many DDR pathway inhibitors are under development.

Indeed, combining ICIs with small molecules, such as cyclin-dependent kinases (CDK) 4/6 inhibitors and poly(ADP-ribose) polymerase (PARP) inhibitors, is an emerging strategy. Trilaciclib, a CDK4/6 inhibitor, is being evaluated within the first-line atezolizumab, carboplatin, and etoposide in a phase II placebo-controlled trial (NCT03041311).

The PARP inhibitor, olaparib, is under investigation in phase II trials, in combination with durvalumab for relapsed SCLC. Although the first phase II study did not meet its primary endpoint (86), this combination of ICI and PARP inhibition is currently being explored (87). Furthermore, the phase III MK 7339-013/KEYLYNK-013 (NCT04624204) is currently ongoing to evaluate the combination of pembrolizumab with concurrent CRT followed by pembrolizumab with or without olaparib in LD-SCLC.

Finally, preliminary results with other targets such as Aurora A kinase inhibitor, CDK7 inhibitors, and epigenetic inhibitors showed modest further benefit in preclinical and clinical models.

The multikinase antiangiogenic anlotinib was also tested in pretreated SCLC and showed a slightly better response compared to placebo (ORR 4.9% vs. 2.6%; DCR 71.6% vs. 13.2%) (88). A higher percentage of responses was reported with the combination of the anti-VEGFR2 apatinib and camrelizumab in the phase II trial PASSION, including both chemosensitive and chemoresistant ED-SCLC (89).

## 5 Conclusions

SCLC is still considered the most aggressive form of lung cancer. However, the advent of immunotherapy has changed the treatment paradigm as well as the outcome of a subgroup of patients affected with extensive SCLC. In this scenario, many open issues remain. Despite the benefit from the combination of ICIs and chemotherapy reported in the recent studies, a significant percentage of patients shows disease progression within 2 years. Moreover, some categories of patients like the elderly or those with an ECOG PS of 2, largely represented in real-world settings, were not studied enough in clinical trials. Therefore, the identification of the predictive factors of the response could be very important in achieving better patient selection in daily clinical practice. Based on recent data from gene profiling and classification in four molecular subtypes of SCLC, as well as the correlation between these molecular subtypes and response to treatment, a strong effort is currently ongoing to personalize cancer care in SCLC tumors, moving this scenario to the new concept of *one-size-does-not-fit-all*. However, we are still far from this concept, and profound knowledge of SCLC cell biology is necessary to improve the survival of these patients.

Besides ICIs combinations, several new treatment strategies, as well as novel molecules to overcome potential mechanisms of resistance, are under investigation with promising results. Thus, is it possible to talk about an effective therapeutic algorithm in SCLC treatment in the near future? Further studies with confirmatory results and a deeper understanding of SCLC biology could be the way to answer this question and expand therapeutic opportunities in this aggressive tumor.

## Author Contributions

LB, JM, and SP conceived the original idea of the article, drafting, and writing the paper. LC, AI, GP, ER, EV and AV revised the scientific content of specific sections of the manuscript and participated in drafting specific section of the paper. LC, AI, GP, ER, EV, AV and JM participated in the critical revision of the paper. LB, JM, and SP participated in the critical revision and editing of the manuscript. LB, LC, AI, GP, ER, EV, AV, JM and SP conceived the original idea and provided critical revision of the manuscript as well as the final approval of the version to publish. All authors contributed to the article and approved the submitted version.

## Conflict of Interest

The authors declare that the research was conducted in the absence of any commercial or financial relationships that could be construed as a potential conflict of interest.

## Publisher’s Note

All claims expressed in this article are solely those of the authors and do not necessarily represent those of their affiliated organizations, or those of the publisher, the editors and the reviewers. Any product that may be evaluated in this article, or claim that may be made by its manufacturer, is not guaranteed or endorsed by the publisher.
